# Chronic Exposure to Field-Level Thiamethoxam Impairs Gut Tissue and Reduces Honeybee (*Apis cerana*) Survival

**DOI:** 10.3390/insects16040372

**Published:** 2025-04-01

**Authors:** Yulong Guo, Changsheng Ma, Wenzheng Zhao, Haiou Kuang, Yakai Tian, Haoyuan Zhang, Yunfei Xue, Hongmei Li-Byarlay, Kun Dong, Xueyang Gong

**Affiliations:** 1 Yunnan Provincial Engineering and Research Center for Sustainable Utilization of Honey Bee Resources, Eastern Bee Research Institute, College of Animal Science and Technology, Yunnan Agricultural University, Kunming 650201, China; guoyulong123@hotmail.com (Y.G.);; 2 Key Laboratory of Pesticide Assessment, Ministry of Agriculture and Rural Affairs, Hunan Academy of Agricultural Sciences, Changsha 410125, China; machangsheng2020@163.com; 3 Yuelushan Laboratory, Changsha 410082, China; 4 Agricultural Research and Development Program, Central State University, Wilberforce, OH 45384, USA; hli-byarlay@centralstate.edu

**Keywords:** *Apis cerana*, thiamethoxam, neonicotinoid insecticides, gut, brain, transcriptome, survival, metabolome

## Abstract

Pesticides such as neonicotinoids frequently harm beneficial insect pollinators and affect their survival, social behavior, digestive system, and metabolism. Investigating the mechanisms behind these impairments is crucial for enhancing pesticide risk assessments. *Apis cerana*, a native honeybee species in Asia, has received limited research attention regarding the toxicological mechanisms of thiamethoxam (TMX) exposure. We exposed newly emerged worker bees of *A. cerana* to a field-relevant dose of TMX (400 ng/g) under laboratory conditions to examine whether TMX exposure triggers similar or distinct effects in different biological processes and tissues. Our results demonstrate that TMX damages the gut cell structure and significantly increases mortality. Gut transcriptomic analysis revealed that the activation of signaling pathways such as glycosphingolipid biosynthesis, Notch signaling, and Wnt signaling likely contributed to structural damage in gut cells. Head transcriptomic results indicated that the activation of pathways including pyruvate metabolism, glycolysis/gluconeogenesis, thiamine metabolism, and riboflavin metabolism might negatively affect the stability of the neural system in *A. cerana*. The metabolic dysfunction of glycine, serine, threonine, as well as glycerophospholipids potentially impairs the neural system, leading to behavioral abnormalities and mortality. In summary, field-level TMX damages the gut cell structure, destabilizes the neural system, and increases the mortality rate of *A. cerana*. These findings demonstrate that TMX exposure induces complex, tissue-specific effects. This study provides a comprehensive understanding of the molecular and physiological impacts of TMX on *A. cerana*, offering valuable insights for the conservation and protection of this important pollinator species.

## Simple Summary

Pollinating insects play a crucial role in maintaining global biodiversity and enhancing crop yield and quality. As a native bee species in China, studying the health of the *Apis cerana (A. cerana)* is of significant value. Thiamethoxam, a neonicotinoid insecticide, poses potential risks to pollinating insects. To explore its impact on the health of *Apis cerana*, we assessed the effects of chronic exposure to field-relevant concentrations of thiamethoxam on gut tissue damage, gene expression changes in gut and head tissues, and metabolic alterations. Our study found that thiamethoxam significantly damaged gut tissue structure, disrupted gene expression in the gut and head, and altered metabolite concentrations in the head, ultimately destabilizing the bee’s nervous system and increasing mortality. In conclusion, thiamethoxam poses a potential hazard to pollinating insects, including the Chinese honeybee. This research provides valuable insights for the protection of pollinating insects and the use of neonicotinoid insecticides.

## 1. Introduction

Honeybees play a crucial role in sustaining the global ecosystem by providing essential services such as pollination, supporting biodiversity, and contributing to food production [1]. Despite their importance, honeybee populations have been in decline worldwide due to several factors. Habitat loss, heavy metal pollution [2,3], the extensive use of pesticides [4], intensive farming practices [3,5] and infections from parasites, bacteria, and viruses [6,7] have all been identified as contributing factors. These threats, either individually or collectively, pose significant risks to the survival and reproductive success of honeybee colonies [8].

Neonicotinoid pesticides are a widely used class of pesticides. Unlike traditional aerial spraying methods, neonicotinoids can be applied through more environmentally favorable approaches, such as seed coating, which minimizes non-target exposure [9]. These insecticides are systemic in nature, allowing them to be absorbed by plants and distributed to various tissues, including leaves, flowers, and pollen, offering comprehensive protection against insect pests [10]. Neonicotinoid insecticides have been widely implicated as a major driver of honeybee colony declines [11,12]. In response to mounting evidence of the harmful effects of neonicotinoids on honeybees, the European Union implemented a temporary ban in 2013 on three of the most commonly used neonicotinoids: clothianidin, imidacloprid, and thiamethoxam [13]. However, residues of TMX are widely detected in nectar and pollen; the average TMX residue in bee food reaches 12.8 ng/g, while in pollen, the average residue reaches 44.9 ng/g, with a maximum of 663.8 ng/g in China [14,15]. Although these field TMX residues do not directly cause bee mortality (TMX LC50 = 8.23 mg/kg for *Apis mellifera* and 16.45 mg/kg for *Apis cerana* [16]), sublethal doses of TMX still affect bee health. Exposure to neonicotinoids has been linked to significant adverse effects in honeybees, including reduced flight and foraging activity [17,18,19], shorter lifespans, weakened immune responses, and impaired pollination service [20,21,22]. It was reported that acute exposure to TMX increased the average homing time of *A. cerana* by approximately 23% and the average flight speed of bees by 27.2%, as well as increased flight time by 18.27% when compared to a control group and flight distance by 46.87% when compared to a control group. Additionally, the study also reported that bees acutely exposed to TMX multiple times showed an average reduction of 36.37% in their learning acquisition and short-term retention abilities. This indicates that acute exposure to field-relevant concentrations of thiamethoxam can significantly impair the learning and memory abilities, flight capabilities, and homing abilities of the Eastern honeybee [19]. However, the effects of chronic TMX exposure on the gut health and survival of *A. cerana* have not been reported to date. Moreover, existing research on TMX and its impact on health has primarily centered around *A. mellifera*. Considering that different bee species exhibit varying sensitivity to pesticides [23,24], these results are not directly applicable to studies on *A. cerana*. Therefore, it is crucial to conduct research on the effects of TMX on *A. cerana*.

The consumption of sugar solution, survival, and hematoxylin and eosin (HE) staining of the gut are often used to assess the effects of pesticides on bee health. For example, when *A. mellifera* bees were exposed to imidacloprid, their sugar solution consumption increased significantly [25]. Meanwhile, exposure of *A. mellifera* to TMX and thiacloprid resulted in a decrease in bee survival [26,27]. However, there are no reports on the effects of TMX on sugar solution consumption, survival, and HE staining in *A. cerana*. Therefore, it is essential to investigate the changes in gut health and survival rates of *A. cerana* after chronic exposure to TMX.

The transcriptome and metabolome are widely applied to elucidate molecular mechanisms underlying pesticide toxicity [28]. TMX acts on the nicotinic acetylcholine receptor (nAChR) [29], disrupting neural signal transmission [30] and altering bee behavior. Imidacloprid caused muscle-related genes to be significantly downregulated, resulting in impaired crawling ability, while also affecting ribosome function and inducing cell dysfunction [25]. Furthermore, it altered the gene expression profile of larvae, triggering premature foraging behavior [31], and decreased worker bees’ body weight and flight ability [18]. It has been reported that a one-fifth LC 50 dose of thiacloprid significantly impaired the learning and memory ability of *A. cerana*. Transcriptome results showed that immune- and stress-related genes were generally upregulated in bees under thiacloprid stress [32]. Exposure to imidacloprid during the queen larval stage of the *A. mellifera* resulted in a significant decrease in larval capping rate and hatching rate. The transcriptome results showed that the upregulated differentially expressed genes were mainly related to chitin binding and calcium ion binding, while the downregulated genes were related to light conduction and visual perception, and studies also have shown that 1 ppb of imidacloprid can also significantly affect the survival rate of queen bees [33]. Researchers found that when *A. cerana* bees were exposed to more than two combinations of three pesticides—imidacloprid, chlorobenzamide, and glyphosate—flight time and distance were significantly reduced, and the transcriptomic and metabolomic results indicate that differentially expressed genes and differentially expressed metabolites are mainly associated with energy-regulated metabolic pathways [18]. However, changes in the transcriptome and metabolome of *A. cerana* following chronic TMX exposure have not been reported.

Therefore, this study exposes *A. cerana* to field-relevant concentrations (400 ng/g) of TMX to observe its effects on sugar solution consumption, survival, and gut cell physiology. Furthermore, transcriptomics and metabolomics are used to identify the potential molecular mechanisms underlying these effects. In summary, our results suggest that chronic exposure to TMX increases the mortality of *A. cerana*, likely by damaging gut tissue structure and impairing the nervous system.

## 2. Materials and Methods

### 2.1. Honeybee Sample Handling and Preparation of Pesticides

All experimental samples were collected from a single colony of *A. cerana* honeybees, consisting exclusively of one-day-old worker bees that had just emerged from their cells. The samples were divided into two groups: an experimental group, which was fed with 400 ng/g of a TMX-supplemented sugar solution for 14 days, and a control group, which was fed with a standard sugar solution (sugar:water = 1:1) for the same duration. Each group contained five replicates, with 15 biological samples per replicate, for a total of 75 honeybees in each group. The feeding experiment was conducted under controlled conditions in an incubator set to 35 °C and 75% relative humidity.

Preparation of the 400 ng/g TMX solution: (1) preparation of a 50% sucrose solution: dissolve 180 g of sucrose in 180 g of water to obtain 360 g of 50% sucrose solution. (2) Preparation of TMX stock solutions: measure 0.012 g of TMX and dissolve it in 1 mL of DMSO to obtain the “Stock 1” solution. Take 100 µL of “Stock 1” and dilute it with 100 g of distilled water to obtain “Stock 2”. (3) Preparation of a 400 ppb TMX sucrose solution: Add 400 µL of TMX “Stock 2” to 40 g of the 50% sucrose solution to obtain a 400 ng/g (400 ppb) TMX sucrose solution.

### 2.2. Sugar Solution Consumption and Survival

The daily sugar solution consumption of worker bees in both the experimental (treatment) and control groups was recorded. The consumption was calculated as the difference between the total amount of sugar solution provided on the previous day and the remaining amount on the current day. This difference was then divided by the number of worker bees to determine the per-bee daily sugar solution consumption. Simultaneously, the number of dead worker bees was counted every morning at a fixed time (10:00 AM) to facilitate the construction of survival curves. The analysis of survival curves used the Kaplan–Meier method and log-rank test to compare survival probability trends over time between the thiamethoxam-exposed group and the control group. These two procedures were conducted daily for 14 days, continuing until the feeding experiment was completed.

### 2.3. Samples for Transcriptome and Metabolome

After 14 days of the feeding experiment, samples were collected for the transcriptome and metabolome. For transcriptomic analysis and RNA-seq, the head issues and the whole gut tissues of worker bees were used, while additional head tissues were used for the metabolome. The dead bees were removed daily to avoid negative effects.

To meet the required input for sequencing, pooled sampling was performed. For transcriptomic sequencing, two head tissue samples were pooled to constitute one biological replicate. For gut tissues, one gut was used as a single biological replicate. For the metabolome, three head tissues were mixed for each biological replicate. The treatment (TMX) group and the control group have eight replicates, respectively. All collected biological samples were placed in sterile 1.5 mL centrifuge tubes and stored at −80 °C until further analysis.

### 2.4. Hematoxylin and Eosin (HE) Staining of the Gut Tissue

To prepare histological sections of worker bee gut tissue for hematoxylin and eosin (HE) staining, the dissected gut tissues were fixed in a paraformaldehyde solution. The tissues were then processed through the HE staining protocol [34]. Following staining, the results were examined under a slide scanning system SQS-12P (40×), and images were captured to document the staining outcomes.

### 2.5. RNA-Seq Sequencing and Transcriptomic Analysis of the Head and Gut

Total RNA was extracted from the tissue using TRIzol^®^ Reagent (Invitrogen, catalog # 15596-026) according to the manufacturer’s instructions. Then RNA quality was determined by a 5300 Bioanalyzer (Agilent, Hangzhou, China) and an ND-2000 (NanoDrop Technologies, Suzhou, China).

RNA purification, reverse transcription, library construction, and sequencing were performed at Shanghai Majorbio Bio-pharm Biotechnology Co., Ltd. (Shanghai, China) according to the manufacturer’s instructions. The sequencing libraries of gut RNA-seq were prepared following Illumina^®^ Stranded mRNA Prep, Ligation (San Diego, CA, USA) using 1 μg of total RNA. After quantification by Qubit 4.0, the sequencing library was prepared on the NovaSeq X Plus platform (Pair end 150 bp) using a NovaSeq Reagent Kit.

The raw paired end reads were trimmed and quality controlled by fastp [35] with default parameters. Then clean reads were separately aligned to reference the genome of *A. cerana* (NCBI: GCF_029169275.1_AcerK_1.0) with orientation mode using HISAT2 [36] software 2.2.1. The mapped reads of each sample were assembled by StringTie [36] using a reference-based approach.

To identify differentially expressed genes (DEGs) between the treatment (TMX) and control groups, the expression level of each transcript was calculated according to the transcripts per million reads (TPM) method. RSEM [37] was used to quantify gene abundance. Essentially, DEG analysis was performed using the DESeq2 [37]. DEGs with |log2FC| ≥ 1 and False Discovery Rate (FDR) < 0.05 were considered to be significantly different between the TMX and control groups. In addition, functional enrichment analysis, including Gene Ontology (GO) and the Kyoto Encyclopedia of Genes and Genomes, (KEGG) was performed via Goatools and Python scipy software 3.8.19, respectively, to identify significant GO terms and metabolic pathways at a Bonferroni-corrected *p*-value < 0.05 compared with the whole-transcriptome background.

### 2.6. Metabolomic Analysis of the Head

A 50 mg sample (8 replicates of pooled heads from the treatment group and control group, respectively) per tube was added to a 2 mL centrifuge tube, to which a 6 mm diameter grinding bead was added. Samples were ground with the Wonbio-96c (Shanghai wanbo biotechnology Co., Ltd., Shanghai, China) frozen tissue grinder for 6 min (−10 °C, 50 Hz), followed by low-temperature ultrasonic extraction for 30 min (5 °C, 40 kHz). The samples were left at −20 °C for 30 min, centrifuged for 15 min (4 °C, 13,000× *g*), and the supernatant was transferred to the injection vial for Liquid Chromatography-Tandem Mass Spectrometry (LC-MS/MS) analysis. As a part of the system conditioning and quality control process, a pooled quality control (QC) sample was prepared by mixing equal volumes of all samples. The QC samples were disposed of and tested in the same manner as the analytic samples of each group (TMX or control).

The LC-MS/MS analysis of the sample was conducted on a Thermo UHPLC-Q Exactive HF-X system equipped with an ACQUITY HSS T3 column (100 mm × 2.1 mm i.d., 1.8 μm; Waters Corporation, Milford, MA, USA) at Majorbio Bio-Pharm Technology Co., Ltd. (Shanghai, China). The metabolites were identified via database searching; the main databases were HMDB (http://www.hmdb.ca/ (accessed on 10 September 2024)), Metlin (https://metlin.scripps.edu/ (accessed on 10 September 2024)) and the self-compiled Majorbio Database (MJDB) of Majorbio Biotechnology Co., Ltd. (Shanghai, China).

The data matrix obtained via database searching was uploaded to the Majorbio cloud platform (https://cloud.majorbio.com (accessed on 11 September 2024)) for data analysis. The R package “ropls” (Version 1.6.2) was used to perform principal component analysis (PCA) and orthogonal least partial squares discriminant analysis (OPLS-DA), as well as 7-cycle interactive validation evaluating the stability of the model. The metabolites with VIP > 1, *p* < 0.05 were determined as significantly different metabolites based on the variable importance in projection (VIP) obtained via the OPLS-DA model and the *p*-value generated by student’s *t*-test.

Metabolites between two groups were mapped onto their biochemical pathways through metabolic enrichment and pathway analysis using the KEGG database (http://www.genome.jp/kegg/ (accessed on 11 September 2024)), based on the principle that annotation analysis of a single metabolite develops into annotation analysis of a group of metabolites. Python packages “scipy.stats” (https://docs.scipy.org/doc/scipy/ (accessed on 11 September 2024)) were used to perform enrichment analysis to obtain the most relevant biological pathways for experimental treatments.

### 2.7. Verification of DEGs Using Real-Time Quantitative PCRs

The expression levels of selected DEGs were confirmed by qPCR in the gut and head tissues of individuals from both the TMX and control groups. The primer pairs evaluated in the study are included in Supplementary Table S1. Total RNA extracted from each bee sample was used in cDNA synthesis for downstream qPCR. The qPCR was performed in a total reaction volume of 20 μL containing the following reagents: 10 μL SYBR Green PCR Master Mix (2×), 2 μL QN ROX Reference Dye, 0.8 μL of each primer (10 μmol/L), 4.4 μL doubly distilled H2O (ddH2O), and 2 μL cDNA. The qPCR reactions were performed on a MX3000P system (Agilent Technologies Inc., Santa Clara, CA, USA) using the following cycle conditions: 95 °C for 3 min, 35 cycles of 95 °C for 30 s, 60 °C for 30 s, and 72 °C for 30 s. A melting curve was observed at the end of each run to confirm the specificity of each primer. Each sample was carried out in triplicate. Each cDNA sample was normalized according to the mRNA level of a housekeeping beta-actin gene. The relative expression levels of the selected DEGs were interpreted by comparative Ct method (2^−ΔΔCt^) [38].

## 3. Results

### 3.1. Honeybee Mortality Rates, Sugar Solution Consumption, and Gut HE Staining

**Figure 1:**
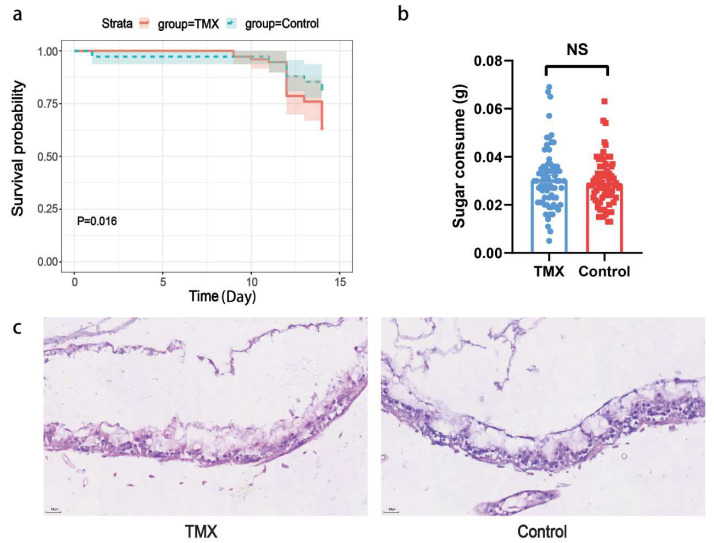
Phenotypic changes in *A. cerana* exposure to TMX. (**a**) Survival curve: The survival rate of *A. cerana* in the experimental group exposed to 400 ppb TMX (red solid line) was significantly lower than that of the control group (green dashed line) (*p* = 0.016). (**b**) Sugar solution consumption: there is no significant difference in daily sugar solution consumption per honeybee (x-axis) between the TMX-exposed group (blue dot plot) and the control group (red dot plot). A Mann–Whitney U test was used to assess significance (N = 70, *p* = 0.4932). NS is short for not significant. (**c**) Image of gut tissue stained with hematoxylin and eosin (HE) staining (40×): gut cells of *A. cerana* in the TMX-exposed group (**left**) and the control group (**right**).

To investigate whether TMX has a significant impact on bee mortality in the experimental and control groups, we recorded the number of dead bees over a 14-day period. Initially, each group (treatment and control) contained 75 bees. The results showed that by day 12, the mortality in both groups began to increase sharply, with the experimental group losing 12 bees and the control group losing 5 bees. On day 14, the experimental group experienced a second peak in mortality, with 10 bees dying, while the control group lost 3. In total, the experimental group had 28 deaths, while the control group had 14. A significant difference in survival curves was observed between the experimental and control groups (*p* = 0.016, Figure 1a).

To investigate whether *A. cerana* exhibits a preference for sugar solution containing TMX, we studied the difference in sugar solution consumption between the experimental and control groups. The results showed no significant difference in the median daily sugar solution consumption per bee between the two groups, suggesting that *A. cerana* may not prefer food containing TMX (Figure 1b).

To investigate whether TMX causes damage to the gut tissues of *A. cerana*, we performed hematoxylin and eosin (HE) staining on the gut tissues of surviving bees from the TMX and control groups after the 14-day feeding trial. The results showed that the gut tissue structure of the TMX-exposed group was damaged to some extent, with incomplete epithelial cell structure in the gut mucosa and a corresponding reduction in gut epithelial thickness. In contrast, the gut epithelial cells of the control group remained relatively intact, with no obvious signs of damage (Figure 1c).

### 3.2. Gut Tissue Transcriptome Analyses

**Figure 2:**
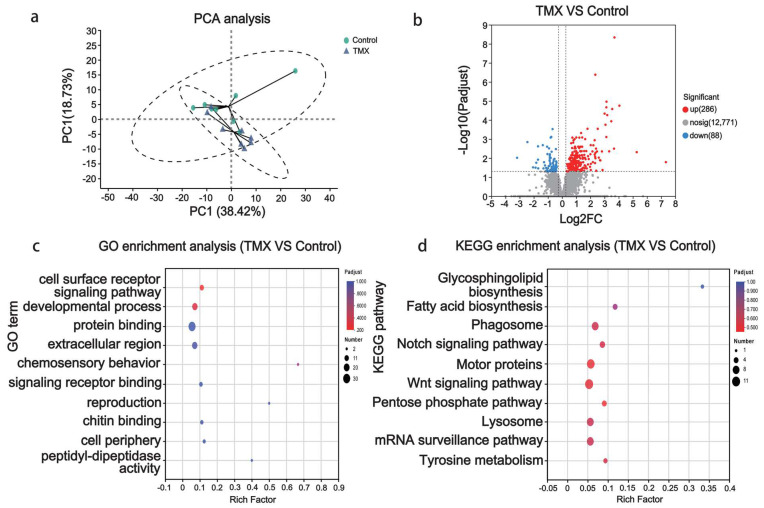
Transcriptomic analysis of gut tissue. (**a**) Principal component analysis (PCA) plot: Blue triangles represent the TMX exposure group and green circles represent the control group. The x-axis represents the first principal component (38.42%), while the y-axis represents the second principal component (18.73%). The circles indicate a 95% confidence interval. (**b**) Volcano plot of differentially expressed genes (DEGs): The x-axis shows the log2 fold change in gene expression between the TMX-exposed group and the control group, while the y-axis represents the −log10 (padjust). The screening criteria for DEGs: padjust < 0.05 and log2|FC| > 1. Red dots represent upregulated genes (286), blue dots represent downregulated genes (88), and gray dots represent non-significant genes (12,771). (**c**) Bubble plot of GO enrichment analysis for DEGs: The x-axis represents the enrichment factor and the y-axis represents the enriched GO terms. The size of the bubbles represents the number of enriched genes. (**d**) Bubble plot of KEGG pathway enrichment analysis for DEGs: The x-axis represents the enrichment factor and the y-axis represents the enriched KEGG pathways. The size of the bubbles represents the number of enriched genes.

To further investigate the molecular-level effects of TMX on the gut tissues of *A. cerana*, we performed transcriptomic sequencing on gut tissues from the TMX and control groups. PCA clustering based on gene expression levels showed a certain degree of overlap between the two groups, with the first principal component explaining 38.42% of the variance between the groups (Figure 2a).

The analysis results of differentially expressed genes (DEGs) revealed that 286 genes were upregulated, 88 genes were downregulated, and the remaining 12,771 genes showed no significant differences compared to the control group (Figure 2b).

To further understand the functions of these DEGs, functional annotation and enrichment analysis were carried out. The results indicated that the top five enriched GO terms affected by TMX are cell surface receptor signaling pathway, developmental process, protein binding, extracellular region, and chemosensory behavior (Figure 2c). The top five KEGG pathways enriched were glycosphingolipid biosynthesis, fatty acid biosynthesis, phagosome, Notch signaling pathway, and motor proteins (Figure 2d) in the gut tissue when bees were exposed to TMX.

Among the top five enriched KEGG pathways, glycosphingolipid biosynthesis, Notch signaling, and Wnt signaling are closely associated with cellular necrosis. In this study, all differentially expressed genes enriched in this pathway were upregulated, such as LOC107998016 (rho-related BTB domain-containing protein 1), LOC107997012 (tyrosine-protein kinase Drl-like), LOC107993075 (protein Wnt-6-like), LOC107996129 (nidogen), and LOC108004272 (disheveled-associated activator of morphogenesis 1). Notably, the LOC107993075 gene encodes the ligand molecule protein Wnt-6-like, which interacts with membrane receptor proteins, while LOC107996129 encodes nidogen, a component of the LRP5/6 receptor. The widespread upregulation of genes in this pathway indicates functional dysregulation, which may contribute to necrosis of gut epithelial cells.

The Notch signaling pathway can indirectly influence necrosis by modulating inflammation and immune responses. The results showed that all differentially expressed genes in this pathway were upregulated, including LOC107995163 (encoding neurogenic locus protein delta), LOC107997670 (encoding protein jagged-1), LOC108002262 (encoding disintegrin and metalloproteinase domain-containing protein 12), LOC107995872 (encoding fringe glycosyltransferase), and LOC107993786 (encoding protein groucho-like). In the pathway relating to glycosphingolipid biosynthesis, the differentially expressed genes LOC10799978 (beta-galactosidase) and LOC108002635 (acetyl-coenzyme A transporter 1) were both upregulated.

### 3.3. Head Transcriptome Analyses

**Figure 3:**
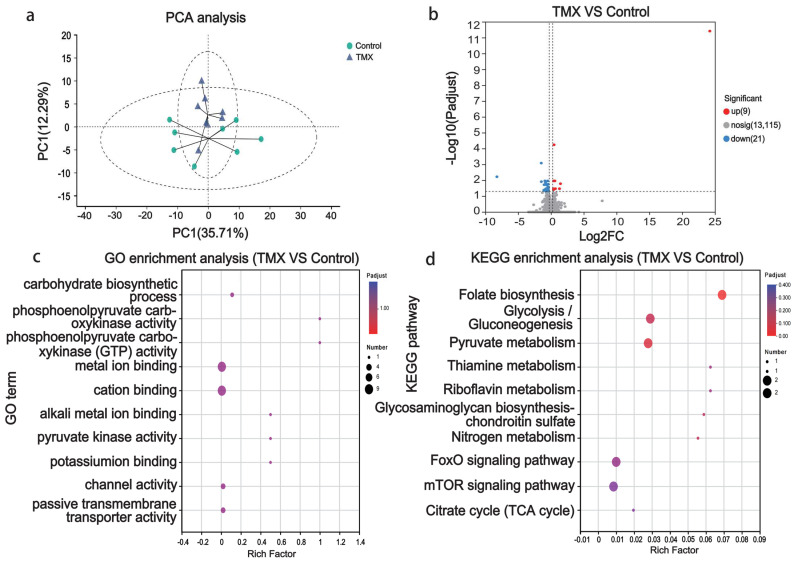
Head transcriptome analysis results. (**a**) Principal component analysis (PCA) plot: The x-axis represents the first principal component (35.71%), while the y-axis represents the second principal component (12.29%). The circles represent a 95% confidence interval. Blue triangles represent the TMX exposure group and green circles represent the control group. (**b**) Volcano plot of differentially expressed genes (DEGs): The x-axis shows the log2 fold change in gene expression between the TMX-exposed group and the control group, while the y-axis represents the −log10 (padjust). The screening criteria for DEGs: padjust < 0.05 and log2|FC| > 1. Red dots represent upregulated genes (9), blue dots represent downregulated genes (21), and gray dots represent non-significant genes (13,115). (**c**) Bubble plot of GO enrichment analysis for DEGs: The x-axis represents the enrichment factor and the y-axis represents the enriched GO terms. The size of the bubbles represents the number of enriched genes. (**d**) Bubble plot of KEGG pathway enrichment analysis for DEGs: The x-axis represents the enrichment factor and the y-axis represents the enriched KEGG pathways. The size of the bubbles represents the number of enriched genes.

The PCA clustering based on the gene expression levels in the head tissues showed a certain degree of overlap between the two groups, with the first principal component explaining 35.71% of the variance between the groups (Figure 3a). Next, we analyzed DEGs between the two groups. The results showed that, compared to the control group, 9 genes were upregulated, 21 genes were downregulated, and the remaining 13,115 genes showed no significant differences (Figure 3b). To further understand the functions of these DEGs, we performed functional annotation and enrichment analysis. The results indicated that the top five enriched GO terms for these DEGs were carbohydrate biosynthetic process, phosphoenolpyruvate carboxykinase activity, phosphoenolpyruvate carboxykinase (GTP) activity, metal ion binding, and cation binding (Figure 3c). The top five KEGG pathways enriched for these DEGs were folate biosynthesis, glycolysis/gluconeogenesis, pyruvate metabolism, thiamine metabolism, and riboflavin metabolism (Figure 3d). Pyruvate metabolism, glycolysis/gluconeogenesis, thiamine metabolism, riboflavin metabolism, citrate cycle, and the mTOR and FoxO signaling pathways are the most relevant to neuronal excitability. These pathways regulate energy production, neurotransmitter release, and synaptic plasticity, all crucial for normal and pathological neuronal activity.

### 3.4. Head Metabolome Analyses

**Figure 4:**
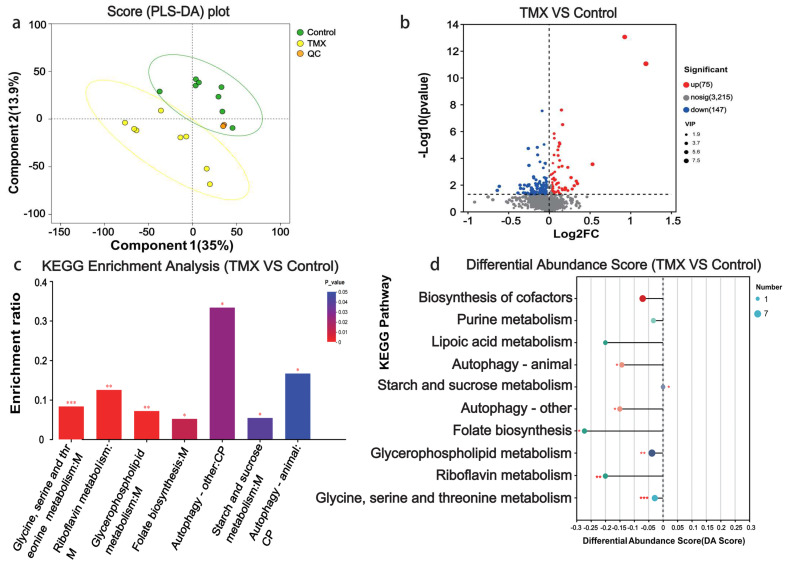
Head metabolome analysis results. (**a**) PLS-DA score plot. The x-axis represents the first principal component (35%), while the y-axis represents the second principal component (13.9%). The circles indicate a 95% PLS-DA confidence interval. Yellow circles represent the TMX exposure group, green circles represent the control group, and orange circles represent the quality control group of a standard sample. (**b**) Volcano plot of differential metabolites (DMs): The x-axis shows the log2 fold change in metabolites between the TMX-exposed group and the control group, while the y-axis represents the −log10 (*p* value). The screening criteria for DMs: *p* < 0.05, VIP (OPLS-DA) > 1. Red dots represent upregulated metabolites (75), blue dots represent downregulated metabolites (147), and gray dots represent non-significant metabolites (3215). (**c**) Bar plot of KEGG pathway enrichment analysis for differential metabolites. The x-axis represents the enriched pathways, while the y-axis represents the enrichment ratio. (**d**) The differential abundance (DA) score plot for KEGG pathways. In the plot, the x-axis represents the differential abundance (DA) score and the y-axis represents the names of KEGG metabolic pathways. The DA score reflects overall metabolite changes in a pathway: a score of 1 means all metabolites are upregulated, −1 means all are downregulated, and the line length represents the absolute value of the score. The size of the dots represents the number of differential metabolites annotated in each pathway. Dots to the right with longer lines suggest upregulation, while dots to the left with longer lines indicate downregulation. * is represent for *p* < 0.05, ** is represent for *p* < 0.01, and *** is present for *p* < 0.001.

We also investigated the effects of TMX on small-molecule metabolites in the head tissues of *A. cerana*. We performed metabolomic sequencing on the head tissues from the experimental and control groups. First, we conducted PLS-DA clustering based on the abundance of metabolites in the head tissues. The clustering results showed that the two groups formed two distinct clusters, with the first principal component explaining 35% of the variance between the two groups (Figure 4a). The results showed that, compared to the control group, 75 metabolites were upregulated, 147 metabolites were downregulated, and the remaining 3215 metabolites showed no significant differences (Figure 4b). To further understand the functions of these differential metabolites, we performed functional annotation and enrichment analysis. The results indicated that the top enriched KEGG pathways for these metabolites were glycine, serine and threonine metabolism, riboflavin metabolism, glycerophospholipid metabolism, folate biosynthesis, autophagy, and starch and sucrose metabolism (Figure 4c). The KEGG pathway differential abundance score plot showed that the significantly enriched KEGG pathways were located on the left side of the central axis, and the overall expression of these pathways tended to be downregulated (Figure 4d).

### 3.5. Quantitative PCR of Candidate Genes of Gut and Head

**Figure 5:**
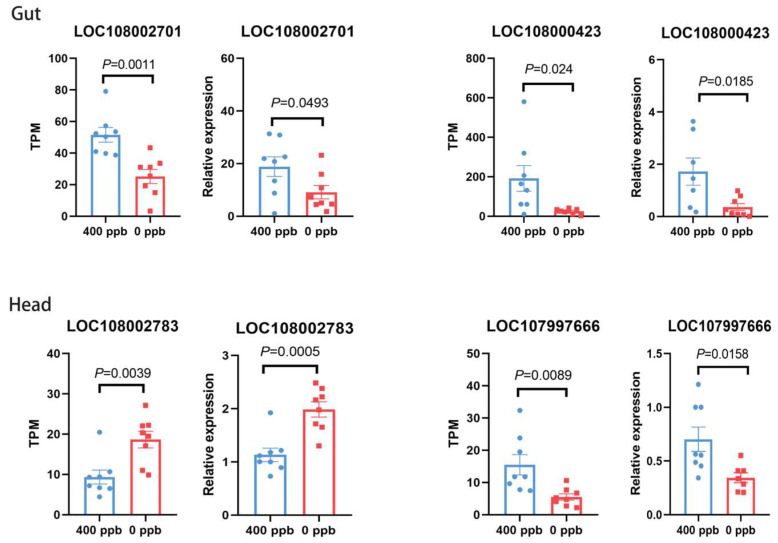
The qPCR verification for the differentially expressed genes of RNAseq data. TPM means transcripts per million reads. The blue represented the treated group, and the red represented the control groups. The validated genes in the gut are LOC108002701 (Sdb) and LOC108000423 (Cyp4g15). The validated genes in the head are LOC108002783 (protein THEM6) and LOC107997666 (proclotting enzyme-like). A *t*-test was used to assess the significance.

To further validate the accuracy of the transcriptome sequencing results, we performed real-time quantitative PCR (qPCR) experiments to verify the expression levels of differentially expressed genes. We validated genes from both the gut and head tissues. The results showed that in the gut tissues, the gene LOC108002701, encoding microtubule-associated protein, was significantly upregulated in both transcriptome sequencing and qPCR experiments (*p* = 0.0011; *p* = 0.0493). Similarly, the gene LOC108000423, encoding cytochrome P450 4g15, was also significantly upregulated in both transcriptome sequencing and qPCR experiments (*p* = 0.024; *p* = 0.0185). In the head tissues, the gene LOC108002783, encoding the protein THEM6, was significantly downregulated in both transcriptome sequencing and qPCR experiments (*p* = 0.0039; *p* = 0.0005). Additionally, the gene LOC107997666, encoding proclotting enzyme-like protein, was significantly upregulated in both transcriptome sequencing and qPCR experiments (*p* = 0.0089, *p* = 0.0158) (Figure 5).

## 4. Discussion

We hypothesize that chronic exposure to TMX will impair the gut tissue and decrease the survival of *A. cerana*. Our results demonstrate that exposure to TMX induces structural damage to gut cells and increases mortality rates in *A. cerana*. Molecular analysis revealed that pathways related to glycosphingolipid biosynthesis, Notch signaling, and Wnt signaling were activated in the gut, suggesting damage to the gut’s cellular structure. In the head, pathways related to pyruvate metabolism, glycolysis, thiamine metabolism, and riboflavin metabolism were disrupted, potentially impairing neural system stability. Additionally, metabolomic analysis identified significant changes in metabolic pathways, further implying that the nervous system was likely disrupted.

Our survival data showed that *A. cerana* mortality from exposure to TMX is much higher than that of the control group, which is consistent with previous research [26]. Prior studies have shown that *A. mellifera* and bumblebees (*Bombus terrestris*) are more likely to consume sugar solutions containing imidacloprid or thiacloprid [39]. Similarly, when *A. mellifera* was exposed to sublethal concentrations of imidacloprid in sugar solution, its consumption of the sugar solution also significantly increased [25]. However, other studies have indicated that the bumblebee species *B. impatiens* did not show a preference for neonicotinoid chemicals and did not exhibit visitation patterns related to floral cues such as color, location, or scent [40]. Our results also indicate that *A. cerana* did not show a preference for sugar solution containing TMX after prolonged exposure to field concentrations. Previous research has shown that low concentrations of thiacloprid and various concentrations of imidacloprid significantly reduce the survival rate of *A. mellifera* [26,27,41]. Similarly, imidacloprid has been shown to interact with the immune system of *B. impatiens,* affecting its survival rate [42]. Our results indicate that the survival rate of *A. cerana* exposed to field concentrations of TMX was significantly lower than that of the control group. This result is consistent with previous studies. All these findings suggest that neonicotinoid insecticides pose a potential threat to pollinating insects.

Our data revealed defects in the structure of gut cells after exposure to TMX, indicating the significant cellular damage caused by this insecticide, which is a new finding in our research. To further investigate the molecular mechanisms of gut cell damage and the high mortality rate in *A. cerana* exposed to field-level TMX, we conducted transcriptomic sequencing of the gut tissues. The transcriptomic analysis revealed that pathways related to cell necrosis were activated in the gut cells. These pathways include glycosphingolipid biosynthesis, phagosome, Notch signaling, and Wnt signaling. Studies have shown that glycoglycerolipids are crucial for the integrity of the cell membrane. Research has demonstrated that glycosphingolipids (GSL) play a key role in maintaining cell membrane integrity, signal transduction, endosome and lysosome function, as well as cell death pathways [43]. The glycosphingolipid biosynthesis pathway indicated that TMX may disrupt the structure of glycosphingolipids outside the cell membrane.

Changes in the phagosome pathway suggest that TMX affects this pathway in the gut. Phagocytes play a crucial role in tissue remodeling and immune defense [44]. Macrophages can clear damaged or dead cells and cellular debris through phagocytosis, with most ingested material being degraded in phagolysosomes [45]. Our results suggest that activation of the phagosome pathway in the cells may be associated with the process of clearing apoptotic and necrotic gut cells. The study suggests that the Notch signaling pathway can promote differentiation, cell death, and autophagy in the hematopoietic system of *Drosophila* [46]. Our results indicate that the Notch signaling pathway was activated in gut cells during the damage process, with the upregulation of many genes in this pathway, which suggests a potential anti-apoptotic role for the cells. Research has shown that the Wnt signaling pathway is an anti-apoptotic signaling pathway [47]. However, in our study, many genes in the Wnt signaling pathway were upregulated, indicating that this pathway is positively regulated, which suggests a potential anti-apoptotic effect in the cells.

Meanwhile, during the feeding process, we observed abnormal behaviors in *A. cerana* prior to death, such as body flipping, abdomen facing upwards, and limb twitching, suggesting that the stability of the bees’ nervous system may have been compromised. Therefore, we conducted transcriptomic sequencing of the head tissues of the surviving bees. The results indicated that differentially expressed genes in the head tissues of bees exposed to field level of TMX were significantly associated with folate biosynthesis, pyruvate metabolism, glycolysis, glycosaminoglycan biosynthesis, thiamine metabolism, and riboflavin metabolism. Previous studies have shown that folate deficiency promotes oxidative damage in the central nervous system (CNS) of apoE-deficient mice [48]. In humans, isolated folate deficiency in the brain can lead to neurocognitive disorders [49]. Our results suggest that the folate biosynthesis pathway may be involved in the abnormal behavioral changes observed in the bees. Researchers have found that pyruvate is a major nutrient in glial cell culture media, and that glial cells play supportive, protective, regulatory, and reparative roles in the CNS [50]. Other research shows that inhibiting glycolysis hampers neuronal energy supply, leading to glutamate receptor overactivation, excitotoxicity, and neuronal death [51]. Our results indicate that the differentially expressed genes LOC107997339 (phosphoenolpyruvate carboxykinase [GTP]-like) and LOC107996114 (pyruvate kinase) are involved in the pyruvate metabolism and glycolysis pathways. These genes were significantly downregulated in the head tissues of bees exposed to field concentrations of TMX, suggesting that TMX exposure may inhibit the pyruvate metabolism and glycolysis processes in the bees’ brain cells. This disruption of energy metabolism likely contributes to abnormal behavior and ultimately results in death.

Additional metabolomic analysis of the head indicated that metabolic processes such as glycine, serine, and threonine metabolism, riboflavin metabolism, and glycerophospholipid metabolism might be associated with damage to the head nervous system. Glycine, serine, and threonine metabolism: glycine (Gly) acts as both an excitatory and inhibitory neurotransmitter in the central nervous system of mammals [52]. Studies in mice show that cystathionine-beta-synthase is vital for CNS development and maintenance [53]. Our results suggest that field-level TMX may have a negative impact on the nervous system of the brain of *A. cerana*, altering the concentrations of molecules such as glycine, cystathionine, and choline. These changes likely affect metabolic processes in the brain, ultimately leading to abnormal behavior. In animal models, riboflavin boosts brain-derived neurotrophic factor gene and protein expression, potentially improving neuro-motor disorders [54]. Our results suggest that field-level TMX may alter the concentrations of several molecules—Lumichrome, Flavin Mononucleotide, and riboflavin—in the riboflavin metabolism pathway in the bees’ brain, thereby likely negatively affecting the metabolic processes of the nervous system. Studies show that changes in brain glycerophospholipid metabolism disrupt neuronal structure and activity, causing abnormal behavior in mice [55]. Our results suggest that changes in the concentrations of molecules such as Phosphatidylethanolamine, Phosphatidylserine, Glycero-phosphoethanolamine, choline, and Phosphatidylcholine may negatively affect the brain’s neurological functions.

The negative impact of TMX exposure on the honeybee host, *A. cerana*, is unquestionable. In this process, the gut microbiota of *A. cerana* have likely been disrupted and significantly altered. These gut microbiota probably play a critical role in maintaining the health and survival of *A. cerana* when exposed to TMX. The diversity and health of gut microbiota are crucial for resilience to external stressors, such as pesticides, viruses, antibiotics, and climate change. Therefore, the changes in the gut microbiota of *A. cerana* exposed to TMX will be a focus of our future research.

## 5. Conclusions

In conclusion, chronic exposure to TMX damages the structural integrity of gut tissue cells in *A. cerana* and significantly reduces the survival rate of bees. Transcriptomic analysis of gut tissues indicates that necrosis-related pathways are activated, which may lead to cell death in the gut. Meanwhile, findings from head transcriptomics and metabolomics collectively suggest that the function of the nervous system in the brain is likely impaired. This impairment leads to behavioral abnormalities in *A. cerana*, ultimately resulting in death. Our findings provide valuable guidance for pesticide usage management and *A. cerana* beekeeping practices.

## Data Availability

Raw sequence data were deposited in the NCBI under BioProject PRJNA1228320. The accession numbers of the raw RNA sequencing data were SRR32551417, SRR32551418, SRR32551419, SRR32551420, SRR32551421, SRR32551422, SRR32551423, SRR32551424, SRR32551425, SRR32551426, SRR32551427, SRR32551428, SRR32551429, SRR32551430, SRR32551431, SRR32551432, SRR32551433, SRR32551434, SRR32551435, SRR32551436, SRR32551437, SRR32551438, SRR32551439, SRR32551440, SRR32551441, SRR32551442, SRR32551443, SRR32551444, SRR32551445, SRR32551446, SRR32551447, and SRR32551448. Total numbers of raw RNA sequencing data were 32.

## Supplementary Materials

The following supporting information can be downloaded at https://www.mdpi.com/article/10.3390/insects16040372/s1: Table S1: Primer sequence; Table S2: Gut DEGs list; Table S3: GO enrichment of gut DEGs; Table S4: KEGG enrichment of gut DEGs; Table S5: Head DEGs list; Table S6: GO enrichment of head DEGs; Table S7: KEGG enrichment of head DEGs; Table S8: KEGG enrichment of head differential metabolites. Figure S9: Expression correlation among biological replicates of the gut; Figure S10: Expression correlation among biological replicates of the head.
