# Supplementary figures and images for: Chronic Exposure to Field-Level Thiamethoxam Impairs Gut Tissue and Reduces Honeybee (Apis cerana) Survival
Source: Insects. 2025 Apr 1;16(4):372. doi: 10.3390/insects16040372 (PMC12028093; doi:10.3390/insects16040372)

## Correlation between samples

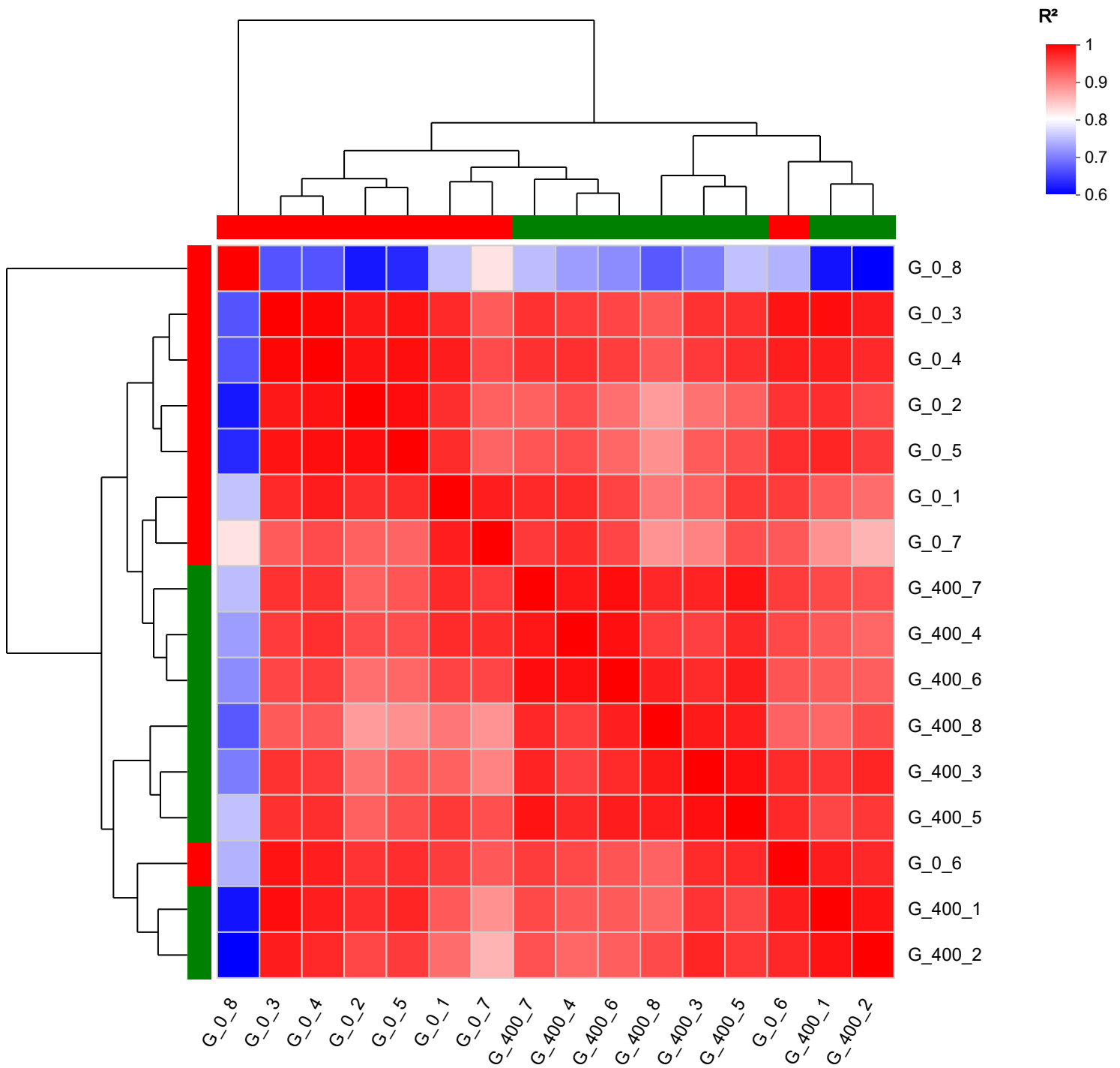

Supplement: Supplementary file 1 [file insects-16-00372-s001.zip › Figure S9 The sample correlation of gut RNAseq data.pdf]

# Correlation between samples

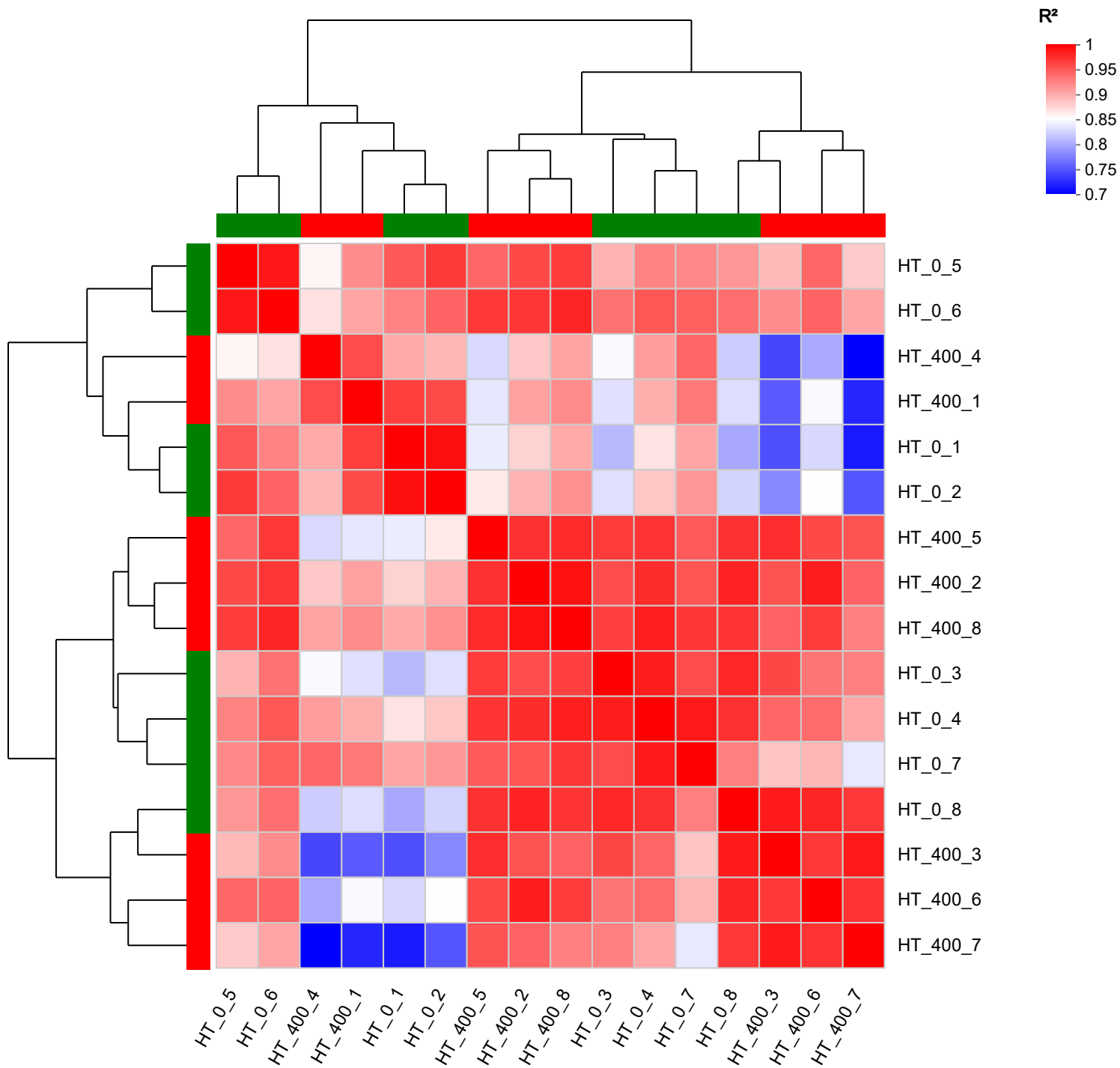

Supplement: Supplementary file 1 [file insects-16-00372-s001.zip › Figure S10 The sample correlation of head RNAseq data.pdf]
